# Experimental Study on the Behaviour of Seismic Actions on a Flexible Glass-Reinforced Plastic Structure Used in Water Transport Pipes

**DOI:** 10.3390/ma14112878

**Published:** 2021-05-27

**Authors:** Ana Diana Ancaș, Ioan Așchilean, Mihai Profire, Florin Emilian Țurcanu, Raluca-Andreea Felseghi

**Affiliations:** 1 Faculty of Civil Engineering and Building Services Engineering, Technical University “Gheorghe Asachi” of Iași, Bd. Dimitrie Mangeron No. 13, 700050 Iaşi, Romania; ancas05@yahoo.com (A.D.A.); profiremihai@yahoo.com (M.P.); 2 Faculty of Civil Engineering, Technical University of Cluj-Napoca, Str. C-tin Daicoviciu, No. 15, 400020 Cluj-Napoca, Romania; andreea.felseghi@ccm.utcluj.ro

**Keywords:** polymer pipeline, GRP, Finite Element Method, seismic safety

## Abstract

This article presents the experimental results obtained by the testing an experimental model of water distribution which is flexible and above-head mounted on a seismic platform, and their validation in a theoretical manner, but also by the Finite Element Method, using the ANSYS simulation program. This type of system shown by the experimental model is desired to be used in practice not only in seismic areas, but also in the areas of heavy road transport, landslides, etc. thorugh the use thereof in the most stressed points of the network (hearth entry/exit, before/after an elbow, etc.) but also on long routes, at optimal distances. The results achieved are related to glass- reinforced plastic (GRP) pipes with a nominal diameter DN = 250 mm, but conclusions may be drawn starting from these to help future research where the mass of the earth is desired to be taken into account. The present results are comprehensive for buried pipes operated dynamically or seismically at low-medium intensity, as this type of earthquake occurs more and more often in Europe. The experimental tests in this article do not have the characteristics necessary for a high intensity seismic action (above 5° Richter).

## 1. Introduction

### 1.1. Background

Under the effect of seismic motion [1], both the earth mass and the underground installations perform oscillating movements as a result of which there can be a significant change in the state of stress and deformations both in the structure and in the earth mass. These changes can cause serious damages that are difficult to remedy and requires significant investment efforts, to which the human and material damages related to the interruption of the water supply system are added, vital in the intervention in case of natural disasters [2]. The severalpossible emergencies but most of all the need to avoid total decommissioning requires compliance with measures and recommendations for the protection of water networks, and among them there is the provision of flexible connections to the pipes, at the exit of a “construction” and on straight lines of 25–30 m.

The issue of the elastic behaviour of underground pipes also arises in the case of flexible pipes made in polymeric materials, while the difficulty in treating the problem and the complex stress of the pipe structure lead to different results from author to author. The calculation of actions must take into account the provisions of the rules and recommendations, but it must be emphasized that an accurate assessment of the intensity of such actions is still a problem that has not been fully solved, the cause being the several factors and parameters involved in the manifestation of the phenomena, the influence of which is difficult to express in analytical relations that are in accordance with reality [3]. In the case of seismic action, these issues become even more complex. The actions in dynamic regime come from the action of the earth (in the case of buried pipes) and from the action of water: the overpressures on the pressure pipes and the effects of the propagation of seismic waves along the structure. Buried flexible and semi-rigid circular pipes have much larger deformations in the cross-section as compared to rigid pipes. The model, in order to express in a satisfactory manner, the behaviour of the real phenomena, must be characterized by as many parameters as possible. On the other hand, the model must be characterized by as few parameters as possible in order to enable the integration of interaction equations [4,5]. All these aspects mean that, at present, there is no exact method to calculate the intensity of earth actions, while the calculation relations used have been obtained based on the processing of the laboratory tests results and observations noted following a practical application.

Practice showed that the adductions and water distribution networks are more vulnerable to seismic action by comparison to other constructions. From the experience in the behaviourof seismic action registered byurban water supply networks, the following more important findings were found [6]:

- the damage of pipes occurs due to crossover breaks, breaks in the rough joint area, breaks in the stiffening areas (manholes, building foundations etc.);
- most of the damages were on the pipes where the route coincided with the dispersion direction of seismic waves, registering damages such as: tearing the ends of pipes from the sockets, destruction of joint parts and crossing cracks;
- the pipes with expansion buffers in the connection points to the rough constructions are more resistant to seismic stresses.

### 1.2. Current State of Research

There are a number of vital infrastructures worldwide whose disruption or destruction would significantly affect the maintenance of vital societal functions, health, safety, security, social or economic well-being of people, and would have a significant impact at local, regional and national level, due to the inability to maintain those functions, while also having similar cross-border effects. These could include cross-border inter-sectorial effects resulting from the interdependent relationships between interconnected infrastructures (e.g., decommissioning of a portion of the drinking water network, making it impossible for fire-fighters to intervene in the event of a disaster, etc.).

Some innovative technology for the design, rehabilitation or modernization of systems infrastructure in order to also includes a way to increase the seismic resilience of vital plant infrastructure is urgently required, given the risks of earthquakes associated with pipeline systems: loss of integrity/tightness of all or part of the subcomponents [7]. This would lead to disaster risks, with a major impact on civil protection, on national security or on the security of major objectives.

The introduction of modern trenchless [8] technologies for the rehabilitation and modernisation of building networks has led to an improvement in the performance of the pipeline because the earth is no longer removed along large sections of the system by conventional excavation. The use of backfill is no longer necessary, removing the need for compaction of the foundation soil that has led to an increase in the seismic resilience of the system.

The concept of “resilience” was taken from materials science and adopted in the 2000s in structural engineering and then at the community and global levels. Among the properties of resilience were listed: robustness, redundancy, resource existence and speed [9,10] The notion of “resilience” can be defined by two categories: the first takes into account everything involved in the immediate post-earthquake stage and reflects the time required for recovery, reaching of the initial level of functionality for the system [11,12], whereas the second category analyzes both the post-event and the pre-event occurrences phase.

The elaboration of a program meant to increase the seismic resilience in the areas of interest is based on the observance of the following principles: the flexibility of the system subjected to the seismic action,—the property of bearing admissible damages, without endangering the functionality; system redundancy—the ability to make it possible to replace system components without disrupting functionality; ingenuity—the ability to identify and use effectively available resources, whether they are material or human, and the speed described by the ability to achieve the proposed objectives in a short time [13,14]. On this basis, seismic resilience was defined, in the sense of the capacity or ability of a system to have a low probability of producing a shock—e.g., to prevent the sudden failure of a structure placed in the necessity to absorb such a shock (if the event of, for instance, a sudden reduction in performance) and to return quickly after such a shock to normal performance.

The experience of the last decades has shown that a qualitative leap is needed, given by the new technological systems for controlling seismic performance [3].

Low, medium or high-intensity seismic activity has a destructive effect on existing water supply and distribution networks. In the specialized literature they are included in Class I—Vital Performance Systems whose operation must be uninterrupted in case of a seismic event. Water networks are also essential for the safety of certain essential subsystems in the event of an earthquake (fire-fighting systems, etc.), so as not to cause loss of human lives, to reduce the adverse impact on the environment and to limit the damage caused by fires. This can be achieved by increasing the seismic resilience of water distribution networks [15].

In the referenced literature there are numerous studies on the seismic resilience of civil structures, tanks, chimneys, on increasing the safety degree in case of earthquakes also for this type of structures, but there is very little research related to water distribution infrastructure systems. 

The studies attempt to solve the problem of sizing and checking thepipelines in the stability condition. The pressure of the backfill earth above the pipeline and the reaction of the ground are actions that can alone lead to loss of stability of flexible pipelines. Added to this is the uniform suction produced by the possibility of vacuum in the pipe. The problem of elastic stability of underground pipelines has been addressed by many researchers. The difficulty of dealing with the problem and the complex stress on the structure of the pipelines has led to different results [16,17], but there is currently no common understanding and unity of views in dealing with the problem.

Notable progress has been made in the theoretical resolution of the problem, but of course considering simplifying assumptions. The analysis of the stability of buried pipes in cross-section is done admitting as interaction model the Winkler model [6] but these results could not be validated experimentally.

Existing international studies on the behaviour of networks in case of dynamic activities refer mainly to the action of heavy traffic over the networks and not to seismic actions [18,19].

Statistics and referenced literature [20,21] contain little data on seismic engineering of the pipes for water supply and distribution. In practice, it has been noted that adductions and distribution networks are the most vulnerable to seismic action. Their sensitivity to seismic action is due to the fact that they unfold over large areas, with a great variability of the physical and mechanical characteristics of the soil in which they are located and generally relatively soft soils [22].

The degree of vulnerability of the water supply and distribution networks has led to the adoption of specific strategies for the protection of such structures against seismic action [23,24,25].

Examination of the numerous cases of rupture of the polymer pipes [26,27] which occurred over time shows that only in particular conditions is a pipe subjected only to vertical pressure in the ground. Most often, the observed ruptures are based on other forces, which produce longitudinal stresses of the pipe, such as tension-compression caused by axial forces or longitudinal bending, generated by bending moments and shear forces in the cross-sections of the pipe. These stresses in the cross-sections indicate a bent bar or beam behaviour and result in the appearance of normal axial stresses in the pipe walls [28,29]. One of the major causes that produce a stressed bar or beam behaviour upon axial bending of the pipes is the movement of the ground due to external forces, such as seismic action or other dynamic stresses.

The dynamic analysis in the case of an earthquake of a buried structure for water transport must be both achieved in the cross section of the structure and longitudinally, to the non-synchronous seismic action in parallel to the axis of work [30]. The impossibility of integrating the equations of motion obtained based on the dynamics of continuous environments led to the development of various models and calculation methods to determine the structure response. The most widely used dynamic method is the Finite Element Method.

Buried circular pipes are cylindrical curved shell structural elements, whose length is very large compared to the diameter, as they are continuously supported on a foundation layer and in permanent interaction with the surrounding earth block and the transported fluids [31,32]. There are three categories of behaviour of the buried circular pipes: those of rigid pipes, of semi-rigid pipes and of flexible pipes.

Flexible pipes have large deformations in the cross-section [33]. These deformations are partly prevented by the reaction of the foundation layer and the well-compacted filling soil, with a favourable effect on the size of the stresses in the pipes. Establishing the behavioural category of the pipes with circular section is of great importance to define the calculation models as accurately as possible and determine a state of stress and strain as close as possible to the real one [34,35].

It is easy to observe that by increasing the flexibility index for the pipe both through the material chosen for the pipe structure and through a special system designed for this purpose, the behaviour of the network faced with dynamic actions is radically improved.

### 1.3. Theoretical Considerations Regarding the GRP Pipeline

The material called generically GRP (fibreglass-reinforced polyesters with added sand) belongs to the category of polymers and is made in layers of different components, properly dosed and assembled in a mould. The GRP polymer is used in multiple fields, such as water and sewage networks (pipes, tanks, fittings), automotive and aeronautical industries, as well as the petrochemical, energy (hydro/thermal energy pipelines, wind turbines), and construction industries.

The production of this polymer can be described in a simplified manner as making alternative layers of fibreglass, resins, as well as other components (quartz sand, dyes, etc.), depending on the destination of the finished product.

The most well-known application of this polymer is the GRP pipes or tubes used in the water-sewerage field.

The GRP pipes have a semi-elastic (they inter-work with the soil, transferring part of the loads to it) and anisotropic behaviour. Of all the materials used in water networks, it has the lowest carbon footprint [36].

A pipe as a main element of a water network must have sufficient strength and/or rigidity and, last but not least, stability, in order to be able to perform its intended function in good conditions [37]. In terms of design standards, pipe materials are placed in one of two general classes: rigid or flexible.

GRP polymer pipes are considered as flexible pipes, because they have high flexibility, allowing for elastic deformations of up to 25% in diameter, without affecting the structural frame [38,39]. Dimensioning in the case of flexible pipes was done starting from the stability condition. In case of rough pipes, the stability condition is considered accomplished, the sizing being achieved by passing the strength condition to limit.

The static computational basis for the GRP pipes is their elastic nature, taking into account the coworking of the pipe with the land. The sizing may be made based on the maximum permissible deformation, not based on the maximum permissible effort, as in the case of rough pipes. The elasticity module is closely related to the geometry of pipe, allowing the maintenance of elastic behaviour.

Table 1 shows the mechanical and elastic features of the GRP material for the nominal diameter of the 250 mm pipe. 

Peculiar to this type of pipe, the term of specific rigidity has to be defined. This is a physical characteristic of the pipe, expressed in N/m^2^ and it represents the measure of the resistance to transverse deformation per meter of length, under external load.

### 1.4. Theoretical Considerations Regarding the Seismic Actions

In certain areas, the large movements of the earth associated with an earthquake can be devastating for any construction, including pipelines. These vital areas are those in which large differential movements occur such as a fissure zone, a soil shear plane or transition areas where the pipe enters a structure [40,41].

Also some soils tend to liquefy during seismic vibration and the buried pipeline may rise or tend to float. On the other hand, most buried flexible pipelines can survive an earthquake. A more flexible pipe material, with a more flexible joint, will allow the pipe to follow the movement of the earth without significant damage [42,43].

In areas with a high probability of earthquakes, seismic design is considered for pipes that must perform an essential function (such as providing water for fire protection), or prevent the release of toxic or flammable contents [44,45]. 

A large amount of data on the behaviour of constructions, as well as of the buried pipes, during earthquakes, has been collected in the last 30 years [46,47,48,49] but nevertheless, no seismic safety measures have been established.

The passage of seismic waves through the ground generates compression/stretching and bending stresses in the buried pipe. 

In the case of seismic wave propagation along the water transport structures, it is considered that the wave propagation environment is represented by both the land and the water in the pipeline. Assuming that the seismic displacement function is a harmonic function, longitudinal forces arise that can be stretches or compressions [50,51].

Values of compression limit deformations have been proposed [52,53], in order to avoid blunting the pipe wall, of the order of 0.4 t/DN up to 2.2 t/DN or (1–2)%, where t—pipe wall thickness, DN-nominal diameter.

### 1.5. Scope and Contributions of the Paper

The results presented in this manuscript are part of a more complex study that is the subject of the Patent Application registered at the Official Bulletin of Intellectual Property—Romanian State Office for Inventions and Trademarks having as main claim “seismic protection process of water distribution networks”.

“No dig” technologies (without digging or without open trenches) for the hydro-urban networks rehabilitation are now known and increasingly applied. The disadvantage of these technologies is that, although the respective network is intervened, only its rehabilitation is carried out, but in no way gives the network protection against seismic actions and other dynamic actions occurred as a result of natural or man-made events (heavy traffic loads, excavations, foundations rehabilitation, subsidies, etc.). The developed research aims to increase the operational safety of water distribution networks by proposing a complex process involving seismic protection of the network, thus removing its decommissioning in case of a seismic event and more.

The proposed technical solution has the advantage that it can be achieved along with the rehabilitation of the network section, this type of rehabilitation (without open trenches) is increasingly used and required, especially for the rehabilitation of pipes in historic cities, to protect buildings, historical monuments, parks, so in areas where a seismic protection of the network is needed, to minimize human and material damage in the event of a natural disaster from earthquakes class.

This article presents the results of the research obtained by testing an experimental model on the seismic platform and verifying the results obtained by theoretical and numerical simulation methods. Its aim is to design and validate a technology meant to improve seismic resilience for the GRP 250 mm water distribution pipes.

The experimental model is a system composed of two sections of GRP250 pipes joined by a flexible piece. The flexibility of the system is conferred in addition to the pipe material and the connecting element.

This system can be used in the exits from the hearth of a pipe, on long rectilinear routes, improving the behaviour of the water distribution system as a whole when confronted with dynamic actions due to heavy transport, landslides, low and medium intensity seismic actions.

If until present, depending on the nature of land, considering the geologic and seismologic studies for water adduction and distribution pipes, it was taken into account the burying depth, following the study, by sampling these flexibility increase system and pipe laying depth could be reduced. The economic environment was open to implement the results of research, because by inserting these junctions, the costs of execution work do not increase in terms of the manual labour of work. The fact that these systems can be used for the pipes subject to heavy traffic brings an additional value to the performed work, following that by continuing the research, to bring improvements to the normative deeds in force. 

The study took into account the worst situation, the movements of pipe not being stopped by the earth massif. This case, the damages are due to the dispersion effect of seismic waves in the underground work. However, it is well known that the damages caused by the seismic movement can also lead to the loss of stability by land sliding.

For this purpose, we consider to continue the research, finding a possibility to make the test on the seismic platform for system buried deeper than 1 m (under the frost depth).

## 2. Experimental Design and Method Used

The experimental tests were performed on the existing seismic platform at the Gheorghe Asachi Technical University of Iași, within a research project [53] carried out over a period of 3 months which consisted of subjecting a pipe section-flexible part-pipe section combination from a GRP polymer, used for the distribution of water, to a compressive force parallel to the axis of the pipe that generates stresses similar to those of the passage of a seismic wave [54].

The seismic platform, inaugurated in 2006, has a capacity of 16 tons and offers the possibility of experimental tests on natural scale models for earthquakes. The Faculty of Constructions and Installations, “Gheorghe Asachi” Technical University of Iași is the only educational institution in Romania that can perform experimental tests related to seismic action [55].

**Figure 1:**
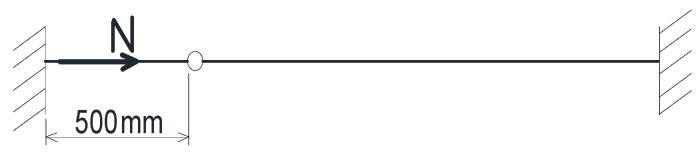
The static schematics of the model.

The static schematics of the model is represented as a doubly embedded bar with an intermediate joint (Figure 1).

**Figure 2:**
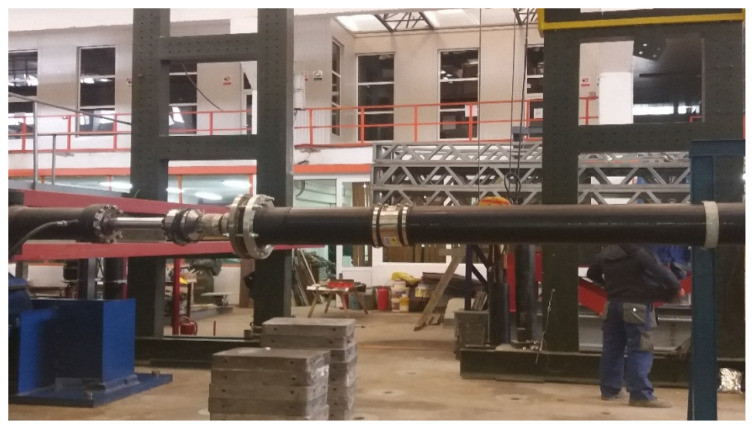
System Consisting of Two Pipeline Sections Joined by Flexseal SC250.

A 500 mm long GRP pipe section was considered, with a diameter of 250 mm, connected by means of the flexible junction to another section of pipe of the same material and of the same diameter, which was fixed so that rotations and displacements in the respective section might be prevented in all three directions (Figure 2). GRP is considered a flexible material, but in order to give greater flexibility to the piping system under test, a Flexal SC250 joint was also used, which accomplished the joint between the 500 mm section and the other fixed section.

The use of a 500 mm section was chosen primarily to enclose the flexible reinforcement as close as possible to the embedment (the exit of a chimney) where the most stressed section occurs. Also the 500 mm section does not allow buckling of the section and the junction.

**Figure 3:**
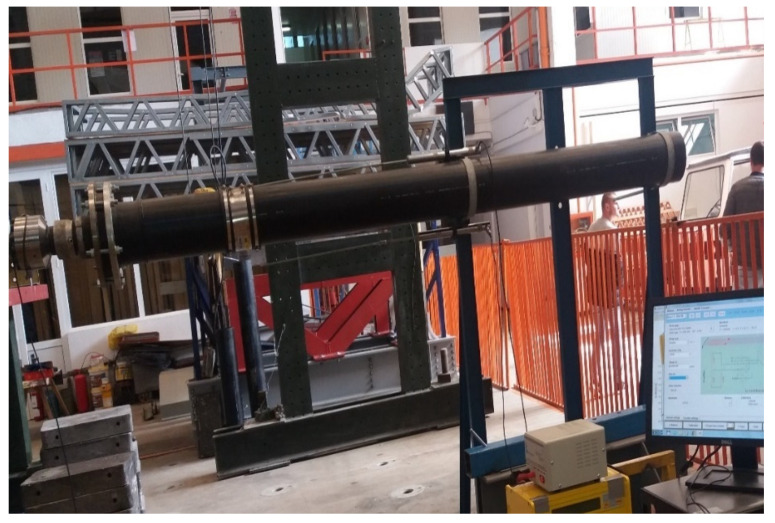
AEP 100 kN Force Cell and Celesco PT5AV Wire Displacement Transducer.

The section was connected through a flange by a horizontal actuator with a capacity of 600 kN. An AEP Transducers force cell with a capacity of 100 kN was mounted in order to measure and record the variations in the force applied to the assembly at the end of the actuator (Figure 3). The lateral pushing force is transmitted to the assembly by means of a joint which, aside the protective role of the equipment used, also has the role of simulating possible rotations that may occur on a structure in case of an earthquake.

The horizontal displacement produced by the actuator was monitored by means of a Celesco PT5AV type wire displacement transducer so that, together with the force value records, it might obtain force-displacement curves as input parameters for evaluating the response of the system which consists of the two pipe sections and the flexible part.

The deformations taken over by the FLEXAL SC250 junction were measured using inductive displacement transducers (LVDT) fixed on a rigid frame and having as measuring points the two ends of the FLEXAL SC 250 (Figure 3).

The entire system was filled with water at a pressure of 1 bar. It should be noted that the Flexseal SC250 flexible sleeve is guaranteed at an internal pressure of 2.5 bar. The water pressure inside the assembly was monitored by means of a manometer mounted on the pipe section that had been fixed on the rigid frame.

The whole system was mounted overhead, this being the most disadvantageous case of analysis of seismic behaviour (all movements occur freely, without being hindered by the existence of soil around the pipe).

During the assembly phase, as well as before the experimental tests, the intention was to ensure the horizontality of the components of the assembly: of the pipe sections, of the connecting sleeve, of the flange for transmitting horizontal loads, as well as for the rods of the inductive displacement transducers and the Celesco PT5AV displacement transducer wire.

The system wasfilled with water at the maximum pressure of 1 bar, although the internal pressure of junction might be 2.5 bars. At pressures higher than 1, the horizontality of the system could not be kept any longer, since the water weight required the additional system. However, we believe that, in the absence of earth around the pipe resisting displacement, even at this low value of pressure, the results obtained can be important for the research.

The experimental data was recorded using a 64-channel ESAM Traveller CF acquisition system. The sampling rate was set at 10 recordings per second on each channel for both the displacement transducers and the force cell.

## 3. Results and Discussion

**Figure 4:**
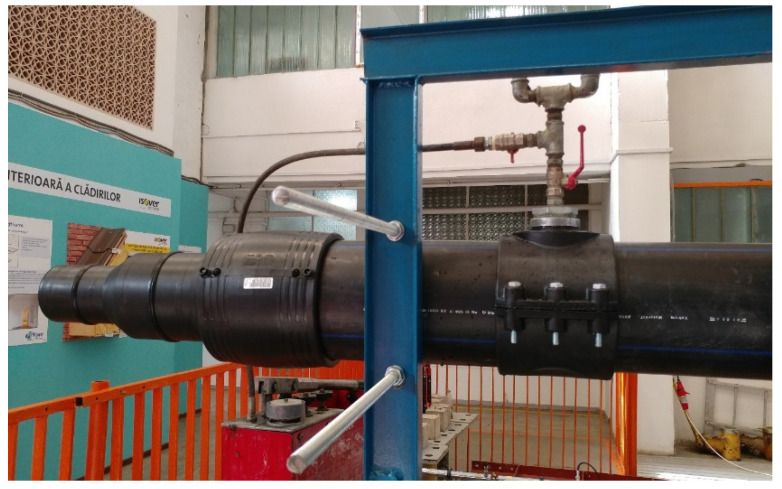
Free end sealing and pressure gauge mounting for internal pressure monitoring.

The experimental scenario was called CA500 (pressure pipe, 500 mm) so that it may reflect the length of the pipe section considered directly attached to a construction, symbolized by the force cell and actuator, as well as the case of the water-filled pipe (Figure 4).

Three loading-unloading cycles were applied aiming to achieve a relative displacement of 20 mm. Around the displacement of 4–5 mm there is a sudden decrease in the value of the applied force which will gradually increase again, until reaching the target value of 20 mm. This sudden decrease in force is due to the rotation of the junction and the rearrangement of the ends of the pipe inside the sleeve (junction). The rotation increases gradually until the lateral displacement target is reached, which leads to the re-positioning of the pipe ends. This is marked by the unconventional shape of the force-displacement curves.

**Figure 5:**
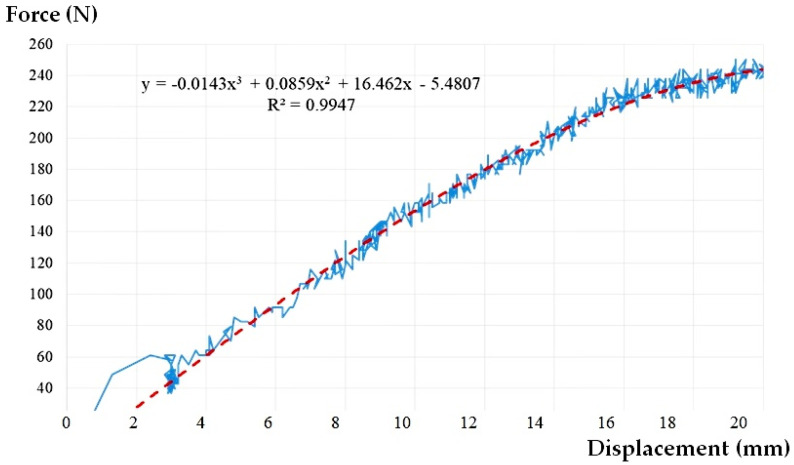
The graph load-displacement curve approximation and for CA500.

The result of the experimental tests for the CA500 scenario is presented in Figure 5.

**Figure 6:**
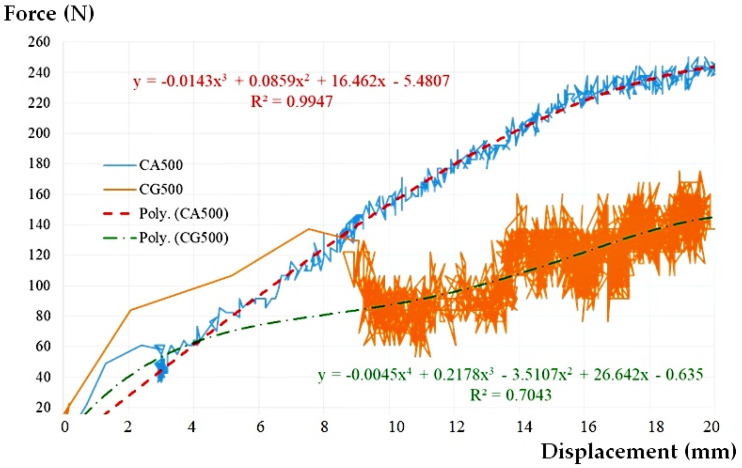
The graph load-displacement curve approximation and cases CG500 and CA500.

It may be seen that the presence of water inside the assembly plays a beneficial role. The water provides stability to the pipe, the force-displacement chart is not chaotic anymore, the magnitudes being much less than the case of empty pipe (Figure 6). Even though this time a rearrangement of the assembly due to the rotation of the flexible sleeve takes place at a lateral displacement of about 3 mm, the shape of the force-displacement curve is a clearer one, without large variations in relation to the midline. This is also reflected by the value of the correlation coefficient R^2^ (see Table 2).

Taking into account the previous research on the CG500 empty pipe [50], a comparative force-lateral displacement graph was made for the 500 mm pipe section, the CG500 and CA500 scenarios presented in Figure 6, and the global results in Table 2.

The contribution of the presence of water to the stability of the whole assembly can be considered as a beneficial one, both regarding the intensity of the force corresponding to the target lateral displacement (increase of 42.61%) and regarding the rotation angle (increase of only 20.17%). According to the table above, at a force of 250 N, the deformation is 2 cm.

It should also be noted that the pressure inside the assembly increased during the experimental test from the initial value of 1 bar to 2 bar, very close to the upper limit of the operating pressure for Flexseal SC200, that is 2.5 bar.

## 4. Results Validation

### 4.1. Validation of the Results in a Theoretical Manner

The results have been theoretically validated starting from the equation corresponding to the special actions (earthquakes) corresponding to the operation phase of the pipeline that establishes the maximum limit of displacement (∆) for which the pipeline resists the considered load [47]:

(1) $$∆=\frac{\mathrm{DL}}{2\left(\mathrm{DN}-t\right)}\leq 5\%$$

where DN, t—nominal diameter (mm), namely the pipe wall thickness (mm).

According to the experimental data centralized in Table 2:

(2) $$∆=\frac{20.20}{2\left(250-6.2\right)}=0.041\leq 5\%$$

According to Figure 5:

(3) $$∆=0.05=\frac{\mathrm{DL}}{2\left(250-6.2\right)}\to \mathrm{DL}=24.38\;\mathrm{mm}\to F>240N$$

At the maximum peak movement, for which the pipe resists to stress, we obtained a value of side movement DL = 24.38 mm, so that according to Figure 6, we obtained a force F>240 N.

According to the aspects above and to the data in Table 2, the water presence in the pipe (pressured system) has a beneficial role, since the system can absorb forces higher than those possible in case of a system without water.

### 4.2. Validation of the Results through the Finite Element Method

The most common method of analysis offers dynamic [56,57] opportunities to analyze large enough to study the behaviour of a pipeline system to seismic action is a Finite Element Method (FEM).

**Figure 7:**
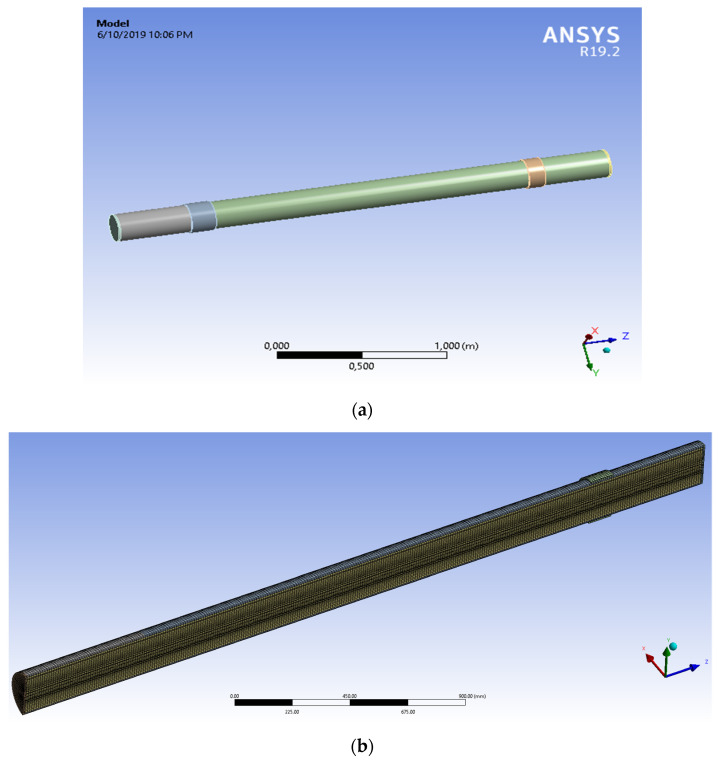
The flexible assembly of GRP pipe (**a**) geometric model (**b**) mesh view.

To validate the experimental results, the electronic application number used to carry out the calculations used in this study was ANSYS R19 [58,59] and the system model is analyzed in accordance with Figure 7.

**Figure 8:**
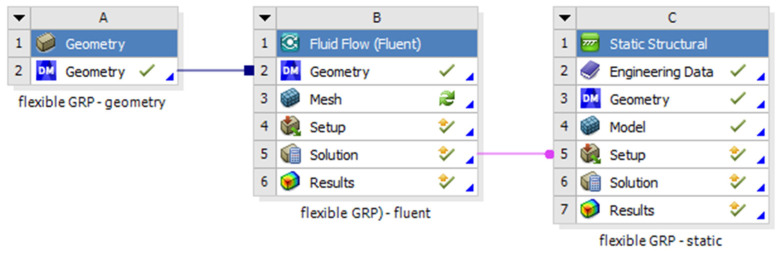
The coupling system.

The geometry of the model was made in spaceclaim 2019 version. For the geometry of the model each dimension was written according to the experimental study. The layers of the pipe were modelled one by one, and the contact area between each layer was set to bonded type, all the materials characteristics are presented in Table 3. The structural analysis was made in static structural model of ANSYS statical 2019 coupled with Fluent resulting a fluid-structured analysis (FSI) Figure 8. From the Fluent model is resulted the pressure given by the water to the pipe walls. In Fluent the viscous model was set to k-epsilon, also the solver was set to a pressure-based type Table 3 and Table 4. The analysis was made in steady state time. The solution method scheme was coupled as the pressure was completed by a static action in static structural module.

Geometry and characteristics of system are presented in Table 3.

**Figure 9:**
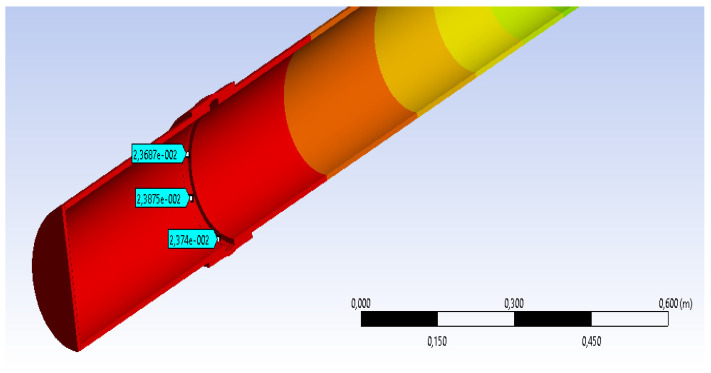
The value of displacements in the most stressed section model CA500, 250 N by MEF-with flexible junction.

The results of the measurement of strains in different points are very close to measurements made experimentally (see Figure 9).

DL max = 23.687 mm

Table 5 shows the parallel between the results obtained experimentally and those obtained by numerical simulation. At the section points, considered at the flexible junction level, it is noticed that the values of side movements are close, so that between the maximum movement experimentally obtained there is a difference of about 3 mm towards the value obtained by MEF. It must considered that the accuracy of results by a numerical method depends on the meshing method and the size of meshing area. However, the results are very close and we can consider that a validation of results experimentally obtained was achieved by numerical methods. Because no experimental tests were performed on a section of pipe without a flexible junction and, encouraged by the validation of the results, it aimed to measure the displacements in the most stressed section for a model with the same characteristics, but without a flexible junction.

The numerical simulation results were compared to the results obtained in the case of GRP pipe section of the water distribution 3000 mm length, subjected to a compressive eccentric force of 250 N similar to the previous case.

**Figure 10:**
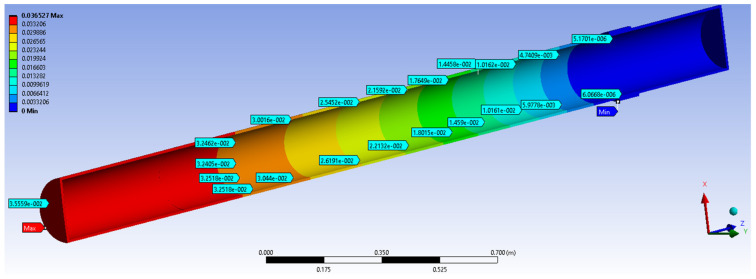
The value of the displacements in the most loaded section model CA500, 250 N, by MEF-without a flexible junction.

Shift values obtained at the same points as in the previous case shown in Figure 10 and are compiled in Table 6.

## 5. Conclusions

Any cause capable of generating states of mechanical stress in a structure constitutes an action on that structure. The action of an earthquake is part of the exceptional actions encountered during the life of a structure with significant values, but a structure is subject to various dynamic actions that can be permanent, temporary or quasi-permanent.

The use of modern materials in the construction of water, sewerage, gas networks, etc. requires also the finding of appropriate solutions that lead to adequate behaviours under the action of various stresses.

One solution for improving the seismic resilience of water supply pipelines is to increase the system flexibility. This can be done by choosing the material, but in many cases that is not enough. On long routes, at the exits from the “construction” it is necessary to increase flexibility because these sections are the most dangerous.

The research herein has demonstrated experimentally and with numerical validation that the use of flexible junctions increases the flexibility of the pipe so that the damages in the most stressed sections appear much more rarely. After the validation in static regime by MEF it is desired to operate the system at different seismic intensities, and to obtain results by numerical methods, this being a subsequent research direction.

The use of the flexible junction allows for the increase of the capable loads taken over by the pipe in a dynamic regime, superimposed on the advantages obtained from the GRP pipe material, a composite material that provides a certain flexibility to it. The obtained results are to be taken into account only in the case of water systems made of this type of composite materials, in the case of rigid pipes the results being insignificant.

The deformations taken over by the FLEXAL SC250 junction were measured using inductive displacement transducers (LVDT) fixed on a rigid frame and with measuring points the two ends of the FLEXAL SC 250.

The study proved experimentally and numerically that the use of a flexible junction, this case FLEXAL SC250 set in the most requested section of the structure, leads to an increase of the capable force. The loading capacity derives from the increase of system flexibility (movement in the same section is lower in the case of the flexible system, at the same value of force compared to the case of system without junction).

It notes that between the values of the displacements of the points in the most loaded section, in the two cases, there is a difference of approx. 10 mm, so that the study can clearly conclude that the use of the flexible junction leads to a better behaviour of the system (Table 6).

The proposed solution can be implemented from the design phase of the water distribution pipes, but it can also be applied in the case of pipe rehabilitation.

This solution is for GRP pipes with a diameter of 250 mm. Experiments are desirable ed to extend the result validation to the case of other pipe diameters, often used in practice.

## Figures and Tables

**Table 1:** Mechanical and Elastic Features of GRP Material.

	Nominal Diameter DN (mm)	Wall thickness t (mm)	Longitudinal Elasticity Module E (MPa)	Transversal Elasticity Module G (MPa)	Poisson’s Coefficient (μ)	Specific Weight (kN/m^3^)	Yield Point (MPa)
GRP250	250	6.2	18,000	3300	0.29	9.5	25

**Table 2:** Comparing experimental results for scenarios CG500 and CA500.

Scenario	Target Lateral Displacement DL (mm)	Force DL (N)	Coefficient of Correlation R^2^	The Maximum Angle of Rotation (°)
CG500	20.02	175.41	0.7043	1.239
CA500	20.20	250.15	0.9947	1.489

**Table 3:** System geometry and characteristics.

Object Name	500/Solid1	FLEXSEAL200/ Solid1	2500/Solid1	FLUSH MOUNTING/Solid1	PLUGS/Solid1	PLUGS/Solid1
	**Material**					
Assignment	GRP	EPDM RUBBER	GRP	Stainless Steel	GRP	GRP
Nonlinear Effects	Yes					
Thermal Strain Effects	Yes					
	**Bounding Box**					
Length X [m]	0.2	0.215	0.2	0.215	0.2	
Length Y [m]	0.2	0.215	0.2	0.215	0.2	
Length Z [m]	0.5	0.15	2.5	0.1	0.01	
	**Properties**					
Volume [m³]	2.2677 × 10^−3^	7.333 × 10^−4^	1.1338 × 10^−2^	4.8886 × 10^−4^	3.1415 × 10^−4^	
Mass [kg]	2.1724	0.7333	10.862	3.7887	2.4346	0.30095
Centroid X [m]	4.7849 × 10^−2^					
Centroid Y [m]	2.1864 × 10^−2^					
Centroid Z [m]	0.25	0.515	1.78	2.58	−5 × 10^−3^	3.035
Moment of Inertia Ip1 [kg·m²]	5.5261 × 10^2^	5.3203 × 10^−3^	5.679	2.3545 × 10^−2^	6.0958 × 10^−3^	7.5351 × 10^−4^
Moment of Inertia Ip2 [kg·m²]	5.5261 × 10^−2^	5.3203 × 10^−3^	5.679	2.3545 × 10^−2^	6.0958 × 10^−3^	7.5351 × 10^−4^
Moment of Inertia Ip3 [kg·m²]	2.0096 × 10^−2^	7.8919 × 10−^3^	9.9769 × 10^−2^	4.0777 × 10^−2^	1.2151 × 10^−2^	1.502 × 10^−3^
	**Statistics**					
Nodes	16.088	5376	79.384	3808	440	454
Elements	2408	728	12,040	504	56	58
Mesh Metric		Aspect ratio 4.23:1.54				
	Skewness Min. 3.5714 × 10^−2^/Max 0.62935/Average 0.35623					
Mesh Type	Hexa-core cell type					
Part Tolerance:	0.00000001					

**Table 4:** The following table defines the steps of the problem and its solution.

Models		
Viscous model		k-epsilon
	k-Epsilon model	standard
	Near-wall treatment	standard wall function
(UAV)	**Boundary Conditions**	
Inlet		Pressure Far-field
	Gauge pressure	2 Bars
	Turbulent intensity	0.9999999776483%
	Hydraulic diameter	0.5 m
Outlet		Pressure outlet
Walls		
	Wall motion	stationary wall
	**Solution Methods**	
Pressure-coupling		SIMPLE
Spatial discretization	Pressure	standard
	Momentum	first-order upwind
	Turbulent kinetic energy	first-order upwind
	Turbulent dissipation rate	first-order upwind
	**Initialization**	
Initialization method		Standard
	Gauge pressure	2 Bars
	Velocity (x,y,z)	(0,0,0) m/s
	Turbulent kinetic energy	0.135 m^2^/s^2^
	Turbulent dissipation rate	0.0465741 m^2^/s^3^

**Table 5:** Comparison of experimental results with results obtained by MEF for CA500, 250N.

Force (N)	Target Lateral Displacement, DL (mm)	
	Experimental Value	Value Obtained with MEF
250	20.22	23.687

**Table 6:** The lateral junction system GA500 vs. system with flexible junction, 250 N.

Measured Points	Horizontal Lateral Displacement	
	Flexible Junction System GA500 (mm)	System without Flexible Junction (mm)
1	23.687	35.116
2	23.875	35.113
3	23.740	33.090
